# Incidence, risk factors, and therapeutic management of equine colic in Lamongan, Indonesia

**DOI:** 10.14202/vetworld.2023.1408-1414

**Published:** 2023-07-09

**Authors:** Faisal Fikri, Dodit Hendrawan, Arya Pradana Wicaksono, Agus Purnomo, Shafia Khairani, Shekhar Chhetri, Salipudin Tasil Maslamama, Muhammad Thohawi Elziyad Purnama

**Affiliations:** 1 Department of Veterinary Science, School of Health and Life Sciences, Universitas Airlangga, Surabaya, Indonesia; 2 Animal Health Division, Indonesian Horse Veterinarian Association, Surabaya, Indonesia; 3 Department of Veterinary Surgery and Radiology, Faculty of Veterinary Medicine, Universitas Gadjah Mada, Yogyakarta, Indonesia; 4 Department of Biomedical Science, Faculty of Medicine, Universitas Padjajaran, Bandung, Indonesia; 5 Department of Animal Science, College of Natural Resources, Royal University of Bhutan, Lobesa, Punakha, Bhutan; 6 Department of Agricultural Biotechnology, Faculty of Agriculture, Eskişehir Osmangazi Üniversitesi, Eskişehir, Turkey; 7 Department of Biology, Graduate School of Natural and Applied Sciences, Eskişehir Osmangazi Üniversitesi, Eskişehir, Turkey

**Keywords:** colic, domesticated animals, lamongan, risk factors, therapeutic management

## Abstract

**Background and Aim:**

Colic is among the common health issues in equine health management. Gastrointestinal (GI) disorders are the most frequent causes of colic, but dysfunction of other organs and systems inside the abdominal cavity may also contribute. Therefore, it is crucial to identify risk factors for colic of specific etiologies. This study aimed to examine the incidence, risk factors, and best therapeutic management practices for horses with colic.

**Materials and Methods:**

A cohort of 256 horses living in Lamongan, East Java, Indonesia, was randomly recruited based on reports of colic symptoms by owners. Diagnosis and treatment were then conducted with the help of owners. Symptom profiles, risk factors, and therapeutic management strategies were analyzed by Chi-square tests.

**Results:**

Of 256 horses enrolled, 217 (84%) were diagnosed with colic, of which 172 (79.3%) were cases of spasmodic colic, 33 (15.2%) of impaction colic, and 12 (5.5%) of intestinal obstruction/displacement. Male sex (χ^2^ = 16.27; p < 0.001), wheat bran feeding (χ^2^ = 15.49; p < 0.001), concentrate feed intake >5 kg/day (χ^2^ = 24.95; p < 0.001), no regular anthelmintic drug treatment (χ^2^ = 67.24; p < 0.001), GI parasite infection (χ^2^ = 65.11; p < 0.001), recurrent colic (χ^2^ = 91.09; p < 0.001), poor body condition score (χ^2^ = 71.81; p < 0.001), limited daily water access (χ^2^ = 127.92; p < 0.001), and indications of dental disease (χ^2^ = 9.03; p < 0.001) were identified as risk factors. The most effective therapies were gastric intubation (χ^2^ = 153.54; p < 0.001), Vitamin B complex injection (χ^2^ = 32.09; p < 0.001), fluid therapy (χ^2^ = 42.59; p < 0.001), and non-steroidal anti-inflammatory drug injection (NSAID).

**Conclusion:**

Colic is highly prevalent among horses in Lamongan, East Java, Indonesia. Proper diet, workload management, regular access to clean drinking water, and dental care can reduce colic risk. Recommended therapies include NSAID injection without other analgesics or spasmolytics, fluid therapy, Vitamin B complex, and gastric intubation.

## Introduction

Diseases manifesting with colic (abdominal pain) are among the most common causes of premature death among horses, and horses are the most frequently afflicted by colic among domesticated animals [1]. The primary causes of colic are illnesses that affect the gastrointestinal (GI) tract, but may occasionally involve other organs inside the abdominal cavity. Primary predisposing factors for equine colic are anatomic abnormalities of the GI system, poor digestive function (from inherent deficiencies or improper diet), and improper management practices by owners [2].

Colic is classified into two types, true colic related to conditions of the digestive tract, and pseudo-colic triggered by other organs in the abdominal cavity. True colic is frequently caused by colon impaction, catarrhal enteralgia, flatulence, and gastric distension. Symptoms may show abrupt onset and both impair performance and lead to behavioral abnormalities. In contrast, pseudo-colic may arise from urolithiasis, liver disease, uterine torsion, renal failure, myositis ossificans, or tying-up syndrome [3]. Severity can fluctuate between mild and severe according to cause and treatment efficacy. Common symptoms of colic (regardless of etiology) include bulimia nervosa, restlessness, excessive sweating, agitation, gazing at the belly, striking, or licking the belly, rolling in the paddock, raking the legs, and twirling [4].

The majority of acute colic cases are successfully treated in <24 h, but a large minority (33%) does not receive a veterinary examination, so the underlying cause may remain untreated. Delay in reporting by the owner to the vet can increase the risk of death in horses with colic [5]. Pain control, GI tract decompression, redressing fluid and biochemical imbalances, and promoting GI motility are the primary aims of colic treatment for horses [6]. However, urgent surgical intervention may be necessary for cases with severe pain unresponsive to these therapies. In addition to distress associated with severe pain, escalating heart rate, deeper peristaltic movements, and hypovolemic shock are important signs of critical colic. Therefore, a thorough evaluation of these indicators is essential [7].

In addition to determining disease history, comprehensive physical examination and evaluation of risk factors are essential for determining the underlying cause and selecting the optimal management for equine colic. Important assessments for diagnosis and prognosis include hydration level, pain intensity, inflammation severity, harm to particular organs, and endotoxemia risk. This study aimed to investigate risk factors and best therapeutic management strategies to improve outcome and reduce colic recurrence.

## Materials and Methods

### Ethical approval and Informed consent

This study did not need ethical approval because it aimed to alleviate animal suffering. However, The Indonesian Horse Veterinarian Association’s standard operating protocols were followed when performing the physical examination and therapeutic management. In addition, written informed consent was obtained from horse owners.

### Study period and location

This study was conducted from October 2020 to April 2022. This study was conducted in Lamongan, East Java, Indonesia, which is located at 6°55’23.6”S 112°24’46.2”E. We considered cases of colics that occur in horses and herds in one stable. The evaluation and therapeutic management of colic were carried out in a timely manner supported by the horses’ owner. Based on the owners’ report, the questionnaire was collected by responding to colic related queries.

### Study design and data collection

This prospective study randomly enrolled 256 horses from Lamongan, East Java, Indonesia, based on owners’ reports of colic. Required study data recorded in the medical report included dates of colic occurrence, detailed clinical signs in the abdominal area, indications of dehydration, presence of intestinal movements, appetite, heart rate, sweating, urination, mucous appearance, body temperature, and classification of colic type. Colic severity was evaluated using the numerical pain scale (NPS) based on clinical signs (Table-1) [8]. Owners also completed a questionnaire on potential risk factors, including age, sex, breed, wheat bran feeding, green fodder feeding, intake of concentrates, use of anthelmintics, presence of GI parasites, recurrent colic history, body condition score (determined according to Carroll and Huntington method), water source, access to drinking water, and presence of dental, and musculoskeletal diseases. Diagnostic aids and supportive care were also used to evaluate suspected recurrent colic episodes and prognosis in each case.

**Table-1:** The NPS for measuring colic in horses.

Pain severity	Clinical signs	Score
Mild	No overt pain behaviors	0
↓↓	Looking at the belly or flank Flehman or lip curling	1
↓↓	Sternal recumbency stretching Restlessness	2
↓↓	Kicking at the belly Pawing	3
↓↓	Attempting to lie down or crouching Lateral recumbency	4
Severe	Rolling	5

Based on the score of the demonstrated clinical signs, a pain score was assigned. If more than one clinical sign was demonstrated, the score was based on the clinical signs with the highest value, NPS=Numerical pain scale

### Treatments

The medical team immediately started therapy if the clinical signs recorded on the medical report indicated colic. In most cases, fluid therapy was initiated by administering lactated Ringers’ solution (Ringer Lactate, Widatra, Indonesia) through the jugular vein. A large volume (around 25 L daily) was administered to treat dehydration and shock, followed by maintenance at 1 L/h. Diarrhea can be difficult to quantify, with some horses losing up to 5 L of fluid per hour. Typically, 2–3 times the maintenance fluid volume was administered to horses with diarrhea, and fluid therapy adequacy was monitored. Concurrently, one of the following non-steroidal anti-inflammatory drugs (NSAIDs) was injected intravenously. Signs of pain were monitored during the treatment period: Flunixin Meglumine (Flumine, Jaapharm, Mano, Singapore) at 1.1 mg/kg body weight (BW), q 12 h, for 3 days; Phenylbutazone (Phenylbute, Phoenix Pharm, USA) once at 4.4 mg/kg BW; ketoprofen (Ketofen, Zoetis, USA) at 2.2 mg/kg BW once daily for 3 days. Vitamin B complex (B-Sanplex, Sanbe, Indonesia) was injected intramuscularly 10 mL/200 kg BW for metabolic stimulation. In some combination therapies, we administered Butylscopolamine (Buscopan Compositum, Boehringer Ingelheim, Germany) at 5 mL/100 kg BW intravenously as a spasmolytic and Xylazine (Virbaxyl, Virbac, France) at 0.2 − 1.0 mg/kg BW intravenously as an opioid [9]. Using a nasogastric tube, we also performed gastric intubation to introduce mineral oil and water as a laxative into the stomach [10].

### Statistical analysis

Symptoms and pain management data were tabulated and expressed as a proportion of the total cohort (%). The questionnaire responses on risk factors were converted into nominal data for analyses. Odds ratio, relative risk, and Chi-square value were calculated, and p < 0.05 was considered statistically significant. All statistical analyses were conducted using SPSS v.25 (IBM, Armonk, NY, USA).

## Results

Of the 256 horses recruited, 217 (84.8%) were diagnosed with colic. Most of these horses exhibited behavioral signs of abdominal pain such as curling the upper lip (92.6%), kicking at the belly (65.9%), looking at the belly (82.1%), pawing at the ground (87.1%), and rolling (75.6%). Physical signs of colic included abdominal distention (17.5%), moderate dehydration (55.8%), constipation (59.5%), intestinal sounds (47.0%), poor appetite (40.5%), profuse sweating (94.6%), frequent urination (88.0%), congested mucous membranes (91.2%), and increased body temperature (88.5%) (Table-2). Evaluation of colic severity using an NPS scale revealed 64 cases of severe colic (75.6%), 40 of moderate colic (18.4%), and 13 of mild colic (6.0%). Diagnostic aids indicated that 172 cases (79.3%) were of spasmodic colic, 33 cases (15.2%) were of impacted colic, and 12 cases (5.5%) were due to intestinal displacement. During the treatment period, we used NSAIDs in 120 cases (55.3%), a combination of NSAIDs and opioids in 95 cases (43.8%), and a combination of NSAIDs and spasmolytics in 2 cases (0.9%). The most frequently employed NSAIDs were flunixin meglumine (93.1%), Phenylbutazone (5.5%), and ketoprofen (1.4%) (Table-3).

**Table-2:** Distribution of clinical signs of colic in horses.

Variables	Colic (n = 217)	Percentage of horses
Abdominal pain		
Curling upper lips up	201	92.6
Kicking at the belly	143	65.9
Looking at the belly	178	82.1
Paw at the ground	189	87.1
Rolling	164	75.6
Abdominal distention		
Absent	179	82.5
Present	38	17.5
Dehydration		
Mild	69	31.8
Moderate	121	55.8
Severe	27	12.4
Intestinal movement		
Absent	37	17.0
Constipation	129	59.5
Diarrhea	51	23.5
Intestinal sound		
Absent	115	53.0
Present	102	47.0
Appetite		
Off food	88	40.5
Poor	85	39.2
Good	44	20.3
Heart rate		
<80 beat/min	98	45.2
>80 beat/min	119	54.8
Profuse sweating	203	93.6
Frequent urination	191	88.0
Congested mucous membrane	198	91.2
Elevated body temperature	192	88.5

n=number of samples

**Table-3:** Evaluation of colic severity, types, and pain management in horses.

Variables	Number of horses	Percentage of horses
Colic severity (n = 217)		
Score 0 (Mild)	0	0.0
Score 1	13	6.0
Score 2	0	0.0
Score 3	40	18.4
Score 4	0	0.0
Score 5 (Severe)	164	75.6
Colic type (n = 217)		
Impaction colic	33	15.2
Spasmodic colic	172	79.3
Intestinal displacement	12	5.5
Pain management (n = 217)		
NSAIDs	120	55.3
NSAIDs, Spasmolytics	2	0.9
NSAIDs, Opioids	95	43.8
NSAIDs type (n = 217)		
Flunixin meglumine	202	93.1
Phenylbutazone	12	5.5
Ketoprofen	3	1.4

n=Number of samples, NSAIDs=Nonsteroidal anti-inflammatory drugs

Chi-square analyses identified male sex (χ^2^ = 16.27; p < 0.001), wheat bran feeding (χ^2^ = 15.49; p < 0.001), feed concentrate intake >5 kg/day (χ^2^ = 24.95; p < 0.001), no regular anthelmintic administration (χ^2^ = 67.24; p < 0.001), GI parasites (χ^2^ = 65.11; p < 0.001), history of colic recurrence (χ^2^ = 91.09; p < 0.001), poor body condition score (χ^2^ = 71.81; p < 0.001), limited daily water access (χ^2^ = 127.92; p < 0.001), and dental disease (χ^2^ = 9.03; p < 0.001) as risk factors. In contrast, colic was not significantly associated with age (χ^2^ = 4.32; p > 0.05), breed (χ^2^ = 2.93; p > 0.05), rearing season (χ^2^ = 0.36; p > 0.05), green fodder feeding (χ^2^ = 0.23; p > 0.05), water source (χ^2^ = 2.79; p > 0.05), or musculoskeletal diseases (χ^2^ = 3.07; p > 0.05) (Table-4).

**Table-4:** Evaluation of risk factors for colic in horses.

Variables	Normal (n = 39)	Colic (n = 217)	χ^2^	p-value	OR	RR
Age						
<5 years old	12	45	4.32	0.12	n/a	n/a
5–10 years old	21	106				n/a
>10 years old	6	66				n/a
Gender						
Male	18	168	16.27	0.00***	0.25	0.59
Female	21	49				2.39
Breed						
Sumba	14	109	2.93	0.23	n/a	n/a
Thoroughbred	8	30				n/a
Mixed	17	78				n/a
Season						
Summer	20	100	0.36	0.55	0.81	1.11
Winter	19	117				0.90
Wheat bran feeding						
No	5	101	15.49	0.00***	0.17	0.28
Yes	34	116				1.63
Feeding on green fodders						
No	29	169	0.23	0.63	0.82	0.96
Yes	10	48				1.16
Concentrate feeding						
<5 kg	0	90	24.95	0.00***	n/a	n/a
>5 kg	39	127				1.71
Anthelmintics administration						
No	0	152	67.24	0.00***	n/a	n/a
Yes	39	65				3.34
Gastrointestinal parasites						
Absent	39	67	65.11	0.00***	n/a	3.24
Present	0	150				n/a
Recurrence of colic						
No	30	22	91.09	0.00***	29.55	7.59
Yes	9	195				0.26
Body condition score						
Poor	4	171	71.81	0.00***	0.03	0.13
Good	35	46				4.23
Water source						
Soft water	12	98	2.79	0.09	0.54	0.68
Well	27	119				1.26
Access to water/day						
Once	0	50	127.92	0.00***	n/a	n/a
Twice	3	145				n/a
3 times	24	16				n/a
More than 3 times	12	6				n/a
Dental diseases						
Absent	39	175	9.03	0.00***	n/a	1.24
Present	0	42				n/a
Musculoskeletal diseases						
Absent	39	201	3.07	0.08	n/a	1.08
Present	0	16				n/a

Significant at *p < 0.05, **p < 0.01,

*** p < 0.001. n = number of samples, χ^2^=Chi-square, OR=Odds ratio, RR=Relative risk, n/a=Not applicable

The most effective treatments were gastric intubation (χ^2^ = 153.54; p < 0.001), Vitamin B complex injection (χ^2^ = 32.09; p < 0.001), and fluid therapy (χ^2^ = 42.59; p < 0.001). Injection of NSAIDs as pain management was always performed according to standard protocols for treating horses with indications of colic. Combination analgesic therapy, including NSAIDS with other analgesics or spasmolytics, was found to be no more effective (χ^2^ = 1.18; p > 0.05) during the colic period (Table-5). Therefore, injection of NSAIDs alone is strongly recommended for pain management during colic periods.

**Table-5:** Therapeutic management of colic in horses.

Treatments	Recovered (n = 212)	Died (n = 5)	χ^2^	p-value	OR	RR
Gastric intubation						
No	2	5	153.54	0.00***	n/a	0.01
Yes	210	0				n/a
Administration of Vitamin B complex						
No	8	3	32.09	0.00***	0.03	0.06
Yes	204	2				2.41
Administration of NSAIDs						
No	0	0	n/a	n/a	n/a	n/a
Yes	212	5				n/a
Combination of analgesics						
No	118	4	1.18	0.28	0.31	0.69
Yes	94	1				2.22
Fluid therapy						
No	0	1	42.59	0.00***	n/a	n/a
Yes	212	4				1.25

Significant at *p < 0.05, **p < 0.01,

*** p < 0.001. n = Number of samples, χ^2^=Chi-square, OR=Odds ratio, RR=Relative risk, n/a=Not applicable, NSAIDs=Nonsteroidal anti-inflammatory drugs

## Discussion

Colic is the primary equine health management problem across the globe. Colic is classified as spasmodic, impacted, or obstructive [11]. Consistent with high global prevalence, more than 80% of horses in this study cohort were diagnosed with colic. Abdominal pain, which is frequent but rarely prolonged in healthy animals, is persistent in spasmodic colic due to increased intestinal peristalsis and spasms that compress enteric nerves. Increased peristalsis can also result in diarrhea. The undigestible feed can cause spasmodic colic and abruptly alter feeding [12]. In contrast, impacted colic is characterized by moderate abdominal pain, depression, and constipation, frequently due to the lack of feed, lack of drinking water, fatigue, dental diseases, other illnesses, or prior surgery. Colic usually occurs acutely due to increased stomach volume and may be accompanied by restlessness, complete anorexia, sudden or gradual pain, and vomiting [13]. In more severe cases, lethargy and shock appear to be the dominant symptoms. An obstruction in the intestine may alter intestinal anatomy, creating invaginations, volvuluses, or strangulation, accompanied by progressive pain and can be fatal [14].

In this study cohort, 168/217 colic cases (77.4%) were male, compared to only 49 females (22.6%). Male horses are extensively used for transportation and agricultural work. In contrast, female horses are primarily maintained for breeding [15], suggesting that colic risk is associated with overexertion and variations in feeding schedules. In contrast, colic prevalence was unrelated to age, breed (Sumba, thoroughbred, or mixed), or rearing season. Most colic cases were Sumba horses, as this is the most popular breed in Indonesia [16]. Domesticated on the island of Sumbawa, the Sumba horse is popular because of its exceptional mobility, agility, and ability to thrive in hot tropical climates. In fact, Sumba horses are frequently given as dowry during bridal ceremonies. Furthermore, Sumba horses are highly social animals that can participate in various festivals, equestrian events, and pasola, which promote tourism.

Suboptimal feeding appeared to be the most frequent cause of colic. Colic was observed in 116 horses receiving wheat bran (53.5%) and 127 receiving >5 kg of concentrate feed daily (58.5%). Horses in the growth phase (around 5 years of age), whether male, or female, require high-quality feed ingredients, including protein with balanced amino acids for muscular development, hormone regulation, and metabolic stimulation [17]. A weaned horse may ingest up to 3.5 kg of concentrate with 14%–16% pure protein daily provided that the concentrate constitutes only 6%–7% of total feed [18]. In addition, it should be emphasized that fodder has a 30:70 dry matter to concentrate ratio. At 1 year of age, horses require 13.5% crude protein with an overall green fodder mix of 40:60. In addition, the ratio of green fodder to concentrate consumption was 55:45 at 18 months of age, with an 11.5% decrease in the crude protein balance. At 24 months, crude protein requirements can reach 10%, with a dry matter ratio of 65:35 for green fodder and concentrate intake [19].

In our study, GI parasitic infections were present in 150 horses with colic (69.1%), while 152 horses with colic (70.0%) did not receive regular anthelmintics. Single or combined tapeworm infections can lead to worm infestations. Deworming significantly improves the treatment of mixed infections. Worm infestations arise from variations in resistance and may further weaken the equine immune system [20]. Multiple factors, including dietary habits, enclosure circumstances, the environment, and the use of anthelmintics, can influence resistance. Environmental factors influencing the development of worm larvae in grasslands include climate, soil conditions, and plant species [21]. Presumably, the conditions in Lamongan, East Java, Indonesia, necessitate routine administration of anthelmintics to reduce colic risk. Indeed, prescribing an anthelmintic 2 weeks before examination was found to significantly reduce colic risk regardless of the specific drug used [22].

In this study, 171 of 217 colic cases (78.8%) also had poor body condition scores. Furthermore, poor body condition was associated with prior colic history as most cases (195 of 217, 89.9%) were recurrent. Daily nutrition, physical exercise intensity, and anthelmintic administration have major impacts on different body condition score components [23]. After feeding, horses with a history of colic should receive precautionary measures, including moderate exercise and regular dental and nail care [24].

In our sample, 42 colic cases had dental disease (19.4%). Equine molars are not designed for chewing rough fodder with a high lignin composition, so regular dental examination is necessary [25]. Compared to horses with no no or mild dental diseases, horses with severe dental disease were more likely to have colic during the previous 12 months, consistent with other studies [26]. While we could not establish a causal relationship due to the retrospective study design and timing of the colic episode relative to dental examinations, previous studies have reported that horses with a history of dental disease are more likely to suffer recurrent colic episodes and that those with a history of infrequent dental checks are more susceptible to simple colonic obstruction and distension colic [27]. Horses with a history of quidding were also found to be at an increased risk of large colon volvulus [28]. Therefore, implementing a dental prophylaxis program by qualified veterinary surgeons could help lower colic incidence in this group of horses [29].

Limited access to water can also contribute to colic risk [30]. In this study, horses had access to water once in 50 cases (23%), twice in 145 cases (66%), 3 times in 16 cases (7%), and more than 3 times in only 6 cases (2%) To avoid colic, horses require at least 8 L of water every 6 h. In one study, more than twice as many horses with access to outdoor enclosures had colic than those without access to water while outside [31]. Furthermore, compared to horses who had access to water from an automatic waterer, horses that drank from a bucket, creek, or pond were at greater risk of gastric rupture. In contrast, the method of watering was not significantly related to colic risk in this study cohort.

The present study revealed the efficacy of NSAIDs, fluid therapy, Vitamin B complex, and gastric intubation for improving colic symptoms and reducing recurrence. Pain management and restraint are essential, so pain-related behavior should also be frequently monitored [32]. Most prior studies have found that NSAIDs are the drugs of choice for addressing colic-associated pain. In the present study, flunixin meglumine was the primary agent for pain management due to its superior ability to block visceral pain [33]. In addition, flunixin meglumine controls inflammation and endotoxemia for 8–12 h and thus can be used as the primary agent to treat most simple cases of colic. In fact, a single injection blocks prostaglandin production for 8–12 h [34]. Further, flunixin meglumine is widely available [35], and the less available ketoprofen was no more effective. Thus, ketoprofen or flunixin are the NSAIDs of preference, but the choice has no effect on the outcome. According to previous research, flunixin is the most frequently prescribed NSAID for the treatment of colic, followed by phenylbutazone, meloxicam, and ketoprofen [36].

In addition to pain control using analgesics, colic treatment should achieve decompression through the restoration of regular bowel movements. This aim may be aided by laxatives and enteral fluid therapy through nasogastric intubation. Colic impactions that are hypovolemic and resistant to analgesics and laxatives should be treated with oral or intravenous therapy [37]. Spasmodic colic contributed to most cases and synergistically affected the course of the condition, so most horses did not require intubation. Nasogastric intubation was used, however, in most cases of impaction colic, as previous studies have concluded that direct administration of water and laxatives into the stomach is the most effective method for decompression [38]. Colonic hydration or the provision of medicines like magnesium sulfate that promote the influx of water into the colon lumen will soften colonic contents and help dissolve the impaction [39]. Similarly, any intervention that improves oral water consumption and motility can help resolve impaction. In the present study, horses with colic also received Vitamin B complex administration as a metabolic stimulant and to reduce lactic acid production [40].

## Conclusion

Colic was reported in the majority of horses in Lamongan, Indonesia, mainly spasmodic colic (79.3%), with impaction colic (15.2%), and intestinal displacement (5.5%) less prevalent. We also identified male sex, wheat bran feeding, >5 kg/day concentrate feed ingestion, no anthelmintic administration, GI parasites, history of recurrent colic, poor body condition score, limited daily access to water, and indications of dental disease as risk factors for colic. We recommend NSAID injection, fluid therapy, Vitamin B complex injection, and (or) gastric intubation as the most effective treatments for equine colic.

## Authors’ Contributions

FF, DH, and APW: Conceptualized and designed the study. DH, APW, AP, SC, STM, and MTEP: Estimated and collected samples. FF, DH, APW, AP, SK, SC, STM, and MTEP: Evaluated the questionnaire and observed clinical signs of colic in horses. SK and MTEP: Helped in the data analysis. SK and SC: Helped in the visualization and validation of tables. FF, SC, and STM: Drafted the manuscript. FF, SC, STM, and MTEP: Revised and submitted the manuscript. All authors have read, reviewed, and approved the final manuscript.

## References

[1] Zeder M.A (2012). The domestication of animals-la domestication des animaux. J. Anthropol. Res.

[2] Sarrafchi A, Blokhuis H. J (2013). Equine stereotypic behaviors:Causation, occurrence, and prevention. J. Vet. Behav.

[3] Cook V.L, Hassel D.M (2014). Evaluation of the colic in horses:Decision for referral. Vet. Clin. Equine Pract.

[4] Wild I, Freeman S, Robles D, Matamoros D, Ortiz M, Rodriguez J, Burford J (2021). Owners'knowledge and approaches to colic in working equids in Honduras. Animals (Basel).

[5] Christophersen M.T, Dupont N, Berg-Sørensen K.S, Konnerup C, Pihl T.H, Andersen P.H (2014). Short-term survival and mortality rates in a retrospective study of colic in 1588 Danish horses. Acta Vet. Scand.

[6] Gitari A, Nguhiu J, Varma V, Mogoa E (2017). Occurrence, treatment protocols, and outcomes of colic in horses within Nairobi County, Kenya. Vet. World.

[7] Gleerup K.B, Lindegaard C (2016). Recognition and quantification of pain in horses:A tutorial review. Vet. Educ.

[8] Maskato Y, Dugdale A.H, Singer E.R, Kelmer G, Sutton G.A (2020). Prospective feasibility and revalidation of the equine acute abdominal pain scale (EAAPS) in clinical cases of colic in horses. Animals (Basel).

[9] Purnomo A, Wicaksono A.P, Hendrawan D, Purnama M.T.E (2020). Comparative study of the efficacy of flunixin, ketoprofen and phenylbutazone in delman horses with mild colic. Syst. Rev. Pharm.

[10] Fehr J (2012). Nasogastric Intubation. Practical Guide to Equine Colic.

[11] Ibrahim H.M.M (2014). Oxidative stress associated with spasmodic, flatulent, and impaction colic in draft horses. J. Equine Vet. Sci.

[12] Munsterman A (2017). Gastrointestinal System. Nutritional Management of Equine Diseases and Special Cases.

[13] Agina O.A (2017). Haematology and clinical biochemistry findings associated with equine diseases-a review. Not. Sci. Biol.

[14] Radcliffe R.M, Liu S.Y, Cook V.L, Hurcombe S.D, Divers T.J (2022). Interpreting abdominal fluid in colic horses:Understanding and applying peritoneal fluid evidence. J. Vet. Emerg. Crit. Care (San Antonio).

[15] Taylor W (2017). Horse demography and use in Bronze Age Mongolia. Quat. Int.

[16] Detha A, Sudarwanto M, Latif H, Datta F, Rahayu P (2013). Fractionation and identification antimicrobial activity of Sumba mare milk protein against causative agent of subclinical mastitis. Glob. Vet.

[17] Schiaffino S, Dyar K.A, Ciciliot S, Blaauw B, Sandri M (2013). Mechanisms regulating skeletal muscle growth and atrophy. FEBS J.

[18] Mack J.K, Remler H.P, Senckenberg E, Kienzle E (2014). No effect of moderate or high concentrate allowance on growth parameters in weanling warmblood foals fed late-cut haylage as forage. J. Anim. Physiol. Anim. Nutr.

[19] Mallicote M, House A.M, Sanchez L.C (2012). A review of foal diarrhea from birth to weaning. Equine Vet. Educ.

[20] Smith A.D, Panickar K.S, Urban J.F, Dawson H.D (2018). Impact of micronutrients on the immune response of animals. Annu. Rev. Anim. Biosci.

[21] Nielsen M.K (2016). Evidence-based considerations for control of *Parascaris* spp. Infections in horses. Equine Vet. Educ.

[22] Scantlebury C.E, Archer D.C, Proudman C.J, Pinchbeck G.L (2015). Management and horse-level risk factors for recurrent colic in the UK general equine practice population. Equine Vet. J.

[23] Worku Y, Wondimagegn W, Aklilu N, Assefa Z, Gizachew A (2017). Equine colic:Clinical epidemiology and associated risk factors in and around Debre Zeit. Trop. Anim. Health Prod.

[24] Vilanova X.M, De Briyne N, Beaver B, Turner P.V (2019). Horse welfare during equine chorionic gonadotropin (ECG) production. Animals (Basel).

[25] Ellis A.D, Fell M, Luck K, Gill L, Owen H, Briars H, Barfoot C, Harris P (2015). Effect of forage presentation on feed intake behaviour in stabled horses. Appl. Anim. Behav. Sci.

[26] Hymøller L, Dickow M.S, Brøkner C, Austbø D, Jensen S.K (2012). Cereal starch, protein, and fatty acid pre-caecal disappearance is affected by both feed technological treatment and efficiency of the chewing action in horses. Livest. Sci.

[27] Schoster A, Altermatt N, Torgerson P.R, Bischofberger A.S (2020). Outcome and complications following transrectal and transabdominal large intestinal trocarization in equids with colic:228 cases (2004–2015). J. Am. Vet. Med. Assoc.

[28] Suthers J.M, Pinchbeck G.L, Proudman C.J, Archer D.C (2013). Risk factors for large colon volvulus in the UK. Equine Vet. J.

[29] Salem S.E, Scantlebury C.E, Ezzat E, Abdelaal A.M, Archer D.C (2017). Colic in a working horse population in Egypt:Prevalence and risk factors. Equine Vet. J.

[30] Padalino B, Hall E, Raidal S, Celi P, Knight P, Jeffcott L, Muscatello G (2015). Health problems and risk factors associated with long haul transport of horses in Australia. Animals (Basel).

[31] Freeman D.E, Mooney A, Giguère S, Claire J, Evetts C, Diskant P (2021). Effect of feed deprivation on daily water consumption in healthy horses. Equine Vet. J.

[32] Abdisa T (2017). Review on practical guidance of veterinary clinical diagnostic approach. Int. J. Vet. Sci. Res.

[33] Makra Z, Csereklye N, Riera M.M, McMullen R.J (2021). Effects of intravenous flunixin meglumine, phenylbutazone, and acupuncture on ocular pain scores in the horse:A pilot study. J. Equine Vet. Sci.

[34] Ziegler A.L, Blikslager A.T (2020). Sparing the gut:COX-2 inhibitors herald a new era for the treatment of horses with surgical colic. Equine Vet. Educ.

[35] Dohrmann J, Hildebrand F, Straub J, Wadephul R, Pusterla N, Freise F, Venner M (2022). Equine proliferative enteropathy in weanling foals on a German breeding farm:Clinical course, treatment and long-term outcome. J. Equine Vet. Sci.

[36] Duz M, Marshall J.F, Parkin T.D (2019). Proportion of nonsteroidal anti-inflammatory drug prescription in equine practice. Equine Vet. J.

[37] Desrochers A, White N.A (2017). Diagnostic Approach to Colic. The Equine Acute Abdomen.

[38] Aderinto-Adike A.O, Quigley E.M (2014). Gastrointestinal motility problems in critical care:A clinical perspective. J. Dig. Dis.

[39] Dupont C, Hébert G (2020). Magnesium sulfate-rich natural mineral waters in the treatment of functional constipation-a review. Nutrients.

[40] Ruaux C.G (2013). Cobalamin in companion animals:Diagnostic marker, deficiency states and therapeutic implications. Vet. J.

